# Crimean-Congo Hemorrhagic Fever in Man, Republic of Georgia, 2009

**DOI:** 10.3201/eid1608.100181

**Published:** 2010-08

**Authors:** Khatuna Zakhashvili, Nikoloz Tsertsvadze, Tamar Chikviladze, Ekaterine Jghenti, Marekhi Bekaia, Tinatin Kuchuloria, Matthew J. Hepburn, Paata Imnadze, Alexander Nanuashvili

**Affiliations:** National Center for Disease Control and Public Health, Tbilisi, Georgia (K. Zakhashvili, N. Tsertsvadze, T. Chikviladze, E. Jghenti, P. Imnadze); O. Ghudushauri National Medical Center, Tbilisi (M. Bekaia); I. Javakhishvili Tbilisi State University, Tbilisi (T. Kuchuloria, P. Imnadze); Technology Management Company, Tbilisi (T. Kuchuloria); US Army Medical Research Institute of Infectious Diseases, Fort Detrick, Maryland, USA (M.J. Hepburn); Service of Antimicrobial Chemotherapy of Georgia, Tbilisi (A. Nanuashvili)

**Keywords:** Crimean-Congo hemorrhagic fever, CCHF, Georgia, viruses, vector-borne infections, zoonoses, letter

**To the Editor:** Crimean-Congo hemorrhagic fever (CCHF) virus is widely distributed in the southwestern regions of the former Soviet Union, the Balkans, the Middle East, western People’s Republic of China, and Africa (*1*). Public health officials in the Republic of Georgia have long suspected that CCHF occurs in this country, but laboratory confirmation by using molecular diagnostic techniques has not been possible there until recently.

CCHF virus is primarily transmitted by ticks, but other modes of transmission have been described (*2*). This virus infects humans mainly by the bite of adult *Hyalomma* spp. ticks. Infected sheep and cattle have also been implicated in transmission (*3*). Contact with highly infectious blood from patients has also led to several nosocomial hospital outbreaks, which resulted in the deaths of medical personnel (*4**,**5*). It is estimated that exposure to CCHF virus leads to symptoms in 1 of 5 patients exposed to this virus (*6*). Mortality rates up to 30% have been reported (*7*).

Virus can be isolated from blood of acutely ill patients by cell cultures or by passage through suckling mice. Antigen-detection ELISA is useful for diagnosis, particularly for severe cases (*2*). PCRs may provide additional sensitivity with no loss of specificity. Antibodies are detectable by a variety of methods and generally appear within 5–14 days of disease onset and coincide with clinical improvement. ELISA detection of immunoglobulin M is an established diagnostic method (*2**,**3*). Ribavirin may be effective for treatment of patients with severe CCHF; in vitro, animal, and clinical experience with this drug support its use (*8*). No human or veterinary vaccines against CCHF are currently recommended (none are licensed in the United States). We report a patient in Georgia with CCHF.

The patient was a 30-year-old man who lived in suburban Tbilisi, Georgia. Fever and sore throat without distinguishing characteristics developed in the patient. After 7 days of symptoms, gastrointestinal bleeding, melena, and hematemesis developed. He was admitted to the First City Hospital in Tbilisi, Georgia, on August 25, 2009. He reported frequent fishing in rural areas. The patient lived in a private house on the outskirts of the city that had a yard and vegetation. No specific rodent exposures were noted, and no other travel was reported.

Because his symptoms increased in severity, the patient was transferred to the Ghudushauri National Medical Center in Tbilisi on August 28, 2009. At this time, the patient had a temperature of 38.0°C–38.5°C, decreased consciousness, and hemorrhages primarily on the chest and medial surfaces of the upper extremities (Figure). Prominent hepatomegaly and moderate splenomegaly were observed. Laboratory tests showed pancytopenia with severe thrombocytopenia (thrombocyte count 4.0 × 10^9^ cells/L, erythrocyte count 3.34 × 10^12^ cells/L, leukocyte count 2.92 × 10^9^ cells/L). Neutropenia was also observed (neutrophil count 788 cells/mm^3^), but hematuria was not observed. Creatinine level was within the reference range. Levels of liver transaminases were increased (alanine aminotransferase 3 U/L, aspartate aminotransferase 1,550 U/L). His bilirubin level was 80 mmol/L (direct bilirubin 41 mmol/L). Chest radiograph showed hemorrhagic alveolitis, and gastroduodenoscopy showed erosive duodenitis. The patient began receiving mechanical ventilation at the time of transfer. CCHF was suspected by the infectious diseases physician who was initially consulted on September 3, 2009.

**Figure:**
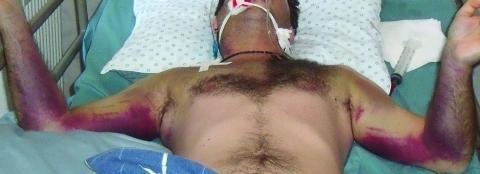
Intubated patient with Crimean-Congo hemorrhagic fever, Republic of Georgia, 2009, showing massive ecchymoses on the upper extremities that extend to the chest.

The National Center for Disease Control and Public Health of **Georgia** investigated the case by obtaining and testing clinical samples. Serum samples obtained on September 4, 2009, were analyzed by using a CCHF IgM ELISA Kit (Vector-Best, Novosibirsk, Russia) and found to be positive for antibodies against CCHF virus (optical density 0.760, cutoff value 0.457). Virus RNA was extracted by using a Mini RNA Extraction Kit (QIAGEN, Hilden, Germany). Samples were positive for CCHF virus by real-time PCR (Roche Diagnostics, Basel, Switzerland) with specific primers (Invitrogen, Carlsbad, CA, USA). The patient was then treated with oral ribavirin (600 mg 3×/d for 14 days), gradually recovered from the infection, and was discharged from the hospital on October 26.

The National Center for Disease Control and Public Health also conducted environmental sampling as part of their case investigation. Rodent brain and lung tissue homogenates were collected from 2 mice captured in the backyard of the patient. Samples were tested by using an antigen detection kit (#97, D-1154; Vector-Best) to confirm the diagnosis. Optical density values were 0.833 and 0.890, respectively (cutoff value 0.334).

This case has serious public health implications for Georgia. For example, laboratory capability to safely detect this virus should be evaluated. Also, healthcare personnel should receive additional education about this disease, particularly so that appropriate precautions can be implemented during initial evaluations. The case was typical of CCHF and showed the pattern of prehemorrhagic, hemorrhagic, and convalescent phases. Hematemesis, melena, and somnolence have been predictors of death in previous investigations (*2*). Frequency of patients with asymptomatic or mildly symptomatic disease should also be determined. Recognition and testing of mild-to-moderate cases may also increase in Georgia as a result of increased awareness in the healthcare community.
